# Synaptic NMDA receptor activity at resting membrane potentials

**DOI:** 10.3389/fncel.2022.916626

**Published:** 2022-07-19

**Authors:** Delia N. Chiu, Brett C. Carter

**Affiliations:** European Neuroscience Institute Göttingen – A Joint Initiative of the University Medical Center Göttingen and the Max Planck Society, Göttingen, Germany

**Keywords:** NMDA receptors, glutamate, somatosensory cortex, hippocampus, postsynaptic signaling

## Abstract

NMDA receptors (NMDARs) are crucial for glutamatergic synaptic signaling in the mammalian central nervous system. When activated by glutamate and glycine/D-serine, the NMDAR ion channel can open, but current flux is further regulated by voltage-dependent block conferred by extracellular Mg^2+^ ions. The unique biophysical property of ligand- and voltage-dependence positions NMDARs as synaptic coincidence detectors, controlling a major source of synaptic Ca^2+^ influx. We measured synaptic currents in layer 2/3 neurons after stimulation in layer 4 of somatosensory cortex and found measurable NMDAR currents at all voltages tested. This NMDAR current did not require concurrent AMPAR depolarization. In physiological ionic conditions, the NMDAR current response at negative potentials was enhanced relative to ionic conditions typically used in slice experiments. NMDAR activity was also seen in synaptic recordings from hippocampal CA1 neurons, indicating a general property of NMDAR signaling. Using a fluorescent Ca^2+^ indicator, we measured responses to stimulation in layer 4 at individual synaptic sites, and Ca^2+^ influx could be detected even with AMPARs blocked. In current clamp recordings, we found that resting membrane potential was hyperpolarized by ∼7 mV and AP firing threshold depolarized by ∼4 mV in traditional compared to physiological ionic concentrations, and that NMDARs contribute to EPSPs at resting membrane potentials. These measurements demonstrate that, even in the presence of extracellular Mg^2+^ and absence of postsynaptic depolarization, NMDARs contribute to synaptic currents and Ca^2+^ influx.

## Introduction

In the mammalian central nervous system, glutamate is the principle neurotransmitter underlying excitatory synaptic signaling (Reiner and Levitz, 2018). Most glutamatergic synapses contain the ionotropic AMPA receptors (α-amino-3-hydroxy-5-methyl-4-isoxazolepropionic acid receptors, AMPARs) and NMDA receptors (N-methyl-D-aspartate receptors, NMDARs; Hansen et al., 2021). AMPARs have relatively low affinity for glutamate, responding rapidly to the brief, high concentration of glutamate present in the synaptic cleft after vesicular release during synaptic activity (Clements et al., 1992) and underlie the most rapid component of synaptic activity. NMDARs have a relatively high affinity for glutamate, slower intrinsic kinetics (Lester et al., 1990), are sensitive to membrane voltage due to extracellular Mg^2+^ block of the NMDAR ion channel (Mayer et al., 1984; Nowak et al., 1984), and have a high permeability to Ca^2+^ (Jahr and Stevens, 1993). One well-studied consequence of these biophysical properties is that the NMDAR contribution to synaptic responses is slower than AMPAR responses, but is enhanced by the coincidence of both glutamate and membrane depolarization.

Coincidence detection is undoubtedly a key function of NMDARs, for example in the context of long-term plasticity induction (Malenka and Nicoll, 1993), but it is not the only one (Dore et al., 2017). Recordings from hippocampus, cortex, and auditory midbrain reveal readily measurable NMDAR-mediated responses (Feldmeyer et al., 2002; Sabatini et al., 2002; Larkum et al., 2009; Oberle et al., 2022), yet it is still often assumed that, in the absence of an explicitly permissive manipulation (e.g., Mg^2+^-free ACSF, postsynaptic depolarization), NMDARs do not contribute to postsynaptic responses to a single synaptic stimulus (Brasier and Feldman, 2008; Larsen et al., 2011, 2014). As the current-voltage relationship of NMDARs at hyperpolarized voltages is known to be non-zero, even with Mg^2+^ present (Mayer et al., 1984; Nowak et al., 1984; Jahr and Stevens, 1990b), synaptic glutamate release should be expected to activate both AMPARs and NMDARs.

Adding another layer of complexity to the issue is the ionic milieu. Recent work has highlighted the fact that cerebrospinal fluid (CSF) contains a higher concentration of K^+^ and lower concentrations of Ca^2+^ and Mg^2+^ than those present in conventional artificial CSF (ACSF; Ding et al., 2016; Rasmussen et al., 2020). What are the implications of more physiological concentrations of these ions on synaptic transmission? Release is Ca^2+^-dependent, and NMDARs are sensitive to Mg^2+^ and voltage, which in turn depends on extracellular K^+^. Because differences in these concentrations are predicted to affect synaptic transmission, but possibly in competing ways, we sought to directly measure their effect on NMDAR function.

In this study, we measured the contribution of postsynaptic NMDARs to synaptic signaling in cortical L4-L2/3 synapses from mouse barrel cortex. We chose to focus on L4-L2/3 because previous work has shown a large NMDAR component of synaptic responses to single APs in paired recordings (Feldmeyer et al., 2002), and because NMDARs play a pivotal role in the synaptic plasticity. Even at the most negative voltages tested, we could detect synaptic NMDAR currents. In ionic conditions mimicking those measured *in vivo* during different behavior states (Ding et al., 2016), postsynaptic NMDAR activity at hyperpolarized potentials was increased relative to traditional ionic conditions. Postsynaptic NMDAR activity could also be detected at single-synapses using two-photon Ca^2+^ imaging, and contributed to postsynaptic potential responses, even at voltages near the neuronal resting potential. NMDAR activity at negative membrane potentials was also measured in hippocampal CA1 synaptic responses to Schaffer collateral stimulation, indicating that although the magnitude of the NMDAR contribution varies among synapse types, this is not a phenomenon restricted to L4-L2/3 synapses.

## Materials and methods

### Animal use

All experiments were performed in accordance with the Institutional Animal Care and Ethics Committees of University of Göttingen (T19.3) and with the German animal welfare laws. Postnatal day 11–24 CD1 mice of both sexes were used for all experiments.

### Slice preparation

Mice were deeply anesthetized with isoflurane and decapitated. The brain was quickly removed into ice cold artificial cerebrospinal fluid (ACSF) consisting of (in mM): 119 NaCl, 4.2 KCl, 1 NaH_2_PO_4_, 25 NaHCO_3_, 10 glucose, 1.2 CaCl_2_, 0.7 MgCl_2_, 1.3 Na-ascorbate, 3 Na-pyruvate. A coronal blocking cut was made and the brain was mounted onto a slicing platform using cyanoacrylate glue (Loctite). The slicing platform was then submerged in continuously bubbled ice-cold ACSF and 270–300 μm slices were made using a vibratome (Leica VT1200S). As slices were cut, they were placed in warm (35°C) ACSF until use.

### Electrophysiological recording

Slices containing primary somatosensory (barrel) cortex or hippocampus were placed in custom-built recording chamber and imaged using an Olympus BX52 upright microscope. Extracellular solution flowed at a rate of 2 ml/min, controlled with a peristaltic pump (Multichannel Systems PPS2). Temperature was maintained using a dual temperature controller (TC-20, NPI) that provided feedback temperature control for an inline heating element (ALA scientific) as well as a resistive heating element embedded in the recording chamber. Measured temperature was 34.7 ± 0.9°C (mean ± standard deviation).

Recording pipettes (PG52151-4, World Precision Instruments) were pulled using a Sutter P-97 puller. Pipette open tip resistance was measured 3.2 ± 1.0 MΩ (mean ± standard deviation) when filled with internal solution. Access resistance (8.0 ± 3.6 MΩ mean ± standard deviation) was estimated in voltage clamp from the current response to a −5 mV test step and was uncompensated, and bridge balance circuitry was engaged in current clamp experiments. Three internal solutions were used. A cesium-based internal was used for most voltage-clamp electrophysiology recordings, and consisted of (in mM): 130 Cs-methanesulfonate, 5 NaCl, 10 HEPES, 5 TEA-Cl, 4 MgCl_2_, 4 Na-ATP, 0.4 Na-GTP, 10 Na-phosphocreatine, 0.1 EGTA; pH 7.35 with CsOH, a measured osmolality of 300 mOsm, and a measured junction potential of −8 mV relative to ACSF. Current-clamp experiments used a potassium-based internal solution consisting of (in mM): 128 K-gluconate, 10 NaCl, 10 HEPES, 4 MgCl_2_, 14 Na-phosphocreatine, 4 Na-ATP, 0.4 Na-GTP, 0.1 EGTA; pH 7.3 with KOH, 300 mOsm, and a junction potential of −11 mV relative to ACSF. For imaging experiments, the same potassium-based internal solution was used, but with EGTA omitted and the addition of (in mM) 0.01 Alexa 594 and 0.3 Fluo-5F (fluorescent dyes from Bio-Techne, Wiesbaden-Nordenstadt, Germany). Voltages are reported with the junction potential corrected.

Stimulating electrodes were made from theta glass capillary tubes (TG150-4, Warner Instrument Corp., Holliston, MA, United States), pulled to have ∼5–20 μm tip diameter, and filled with a HEPES-buffered modified Tyrode’s solution consisting of (in mM): 155 NaCl, 2.5 KCl, 20 HEPES, 1 MgCl_2_, pH 7.4 with NaOH. The stimulating electrode was placed in the visually identified L4 region in the same cortical column as the recorded L2/3 neuron (e.g., Feldman, 2000).

Recordings were made with MultiClamp 700B amplifiers (Molecular Devices), sampled at 50 kHz and filtered at 10 kHz, using either Prairieview 5.4 software (Bruker), or Igor Pro 8 (WaveMetrics) controlling an ITC-18 (HEKA) acquisition interface.

Chemicals were purchased from Carl Roth and Sigma-Aldrich. Pharmacological agents were purchased from Tocris (R-CPP), Hello Bio (NBQX, D-AP5), and Abcam (picrotoxin).

### Two-photon imaging

Two-photon imaging and electrophysiology was performed using a Bruker Ultima In Vitro BX51 system (Bruker). The imaging laser (Coherent Ultra II) was tuned to 810 nm. Fluorescence was separated into red (epifluorescence) and green (epi- and trans-fluorescence) channels and detected with GaAsP PMTs (H7422PA-40 SEL, Hamamatsu). Analysis was performed off-line using Igor Pro 8.

Imaging began at least 20 min after break-in to allow diffusion of fluorescent dyes into the cell. The red fluorescence channel was used to measure morphology (using the Alexa 594 dye) and define the structure(s) of interest. The green fluorescence channel was used to measure Ca^2+^-sensitive fluorescence (using the Fluo-5F dye). After filling, the L2/3 dendritic arbor was searched while stimulating in L4 until a response was seen in the green channel. Active synapses measured had a geometric distance from the soma ranging from 24 to 102 μm (61 ± 27 μm mean ± standard deviation), similar to the distribution measured from electron microscopy reconstructions (e.g., Feldmeyer et al., 2002). Once a response was found, synaptic activity was measured using repetitive scans across the structure with a per-pixel dwell time of 6 μs, and a resulting per line time resolution ranging between 0.5 and 2 ms. Stimulus induced Ca^2+^-sensitive fluorescence was quantified by measuring the change in green fluorescence over time, G(t), relative to the pre-stimulation baseline, G(0), normalized to the red fluorescence signal:


$$\frac{△\,G}{R}=\frac{\left(G\,\left(t\right)-G\,\left(0\right)\right)}{R}$$

### Analysis

Peak synaptic responses were measured relative to the baseline just before stimulation. Paired-pulse measurements were made from the average of 3 to 10 recordings, with the response to a single pulse subtracted from the paired stimulation (to account for any overlapping currents). Decay time course is reported as the weighted tau from a two-exponential fit to the falling phase of the response.

The NMDAR conductance-voltage measurements (Figures 2, 3) were constructed by first estimating the reversal potential for the given ACSF condition, and then converting the peak current to conductance using Ohm’s Law approximation:


$$g_{N\,M\,D\,A}=\frac{i_{N\,M\,D\,A,p\,e\,a\,k}}{\left(V_{h\,o\,l\,d\,i\,n\,g}-V_{r\,e\,v}\right)}$$

The conductance-voltage relationship was then fitted using a sigmoid function (Jahr and Stevens, 1990b):


$$g\,\left(V\right)=\frac{1}{1+a*e^{\frac{-V}{b}}}$$

Resting membrane potential was measured in current clamp recordings with no current injection. The different ACSF solutions were presented in a randomized order (Figure 6). The F-I curves (Figure 6C; action potential firing frequency versus current injection) were constructed by counting the number of action potentials induced by a current injection which was delivered in 20 pA steps. Threshold was measured from as the voltage at which the change in voltage exceeded 10 mV/ms in the first action potential induced by current injection (Bean, 2007).

### Statistics

Data are summarized as mean ± standard error of the mean (SEM) unless otherwise noted. Comparisons between groups were made using non-parametric statistical tests, the Wilcoxon rank-sum test for paired comparisons, and the Mann–Whitney *U* test for unpaired comparisons. Threshold for significance was set at 0.05. For experiments with multiple comparisons, the non-parametric Friedman’s test was used. Friedman’s test statistic is reported with the critical value in parentheses as well as the overall *p* value. If the *p* value was below 0.05, pairwise comparisons were made using the Wilcoxon rank-sum test for paired data or the Mann–Whitney *U* test for unpaired data, and Holm–Sidak correction was used for multiple comparisons.

## Results

### NMDA receptors contribute to excitatory postsynaptic currents

**FIGURE 1:**
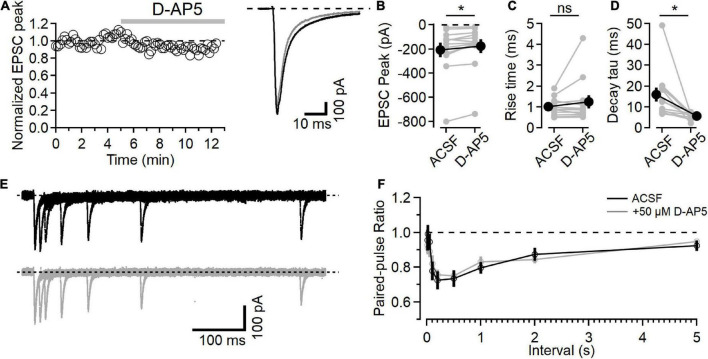
NMDARs contribute to L4-L2/3 EPSCs. **(A)** Left, example time course of EPSC peak measurements in response to bath application of 50 μM D-AP5. Right, average response in control ACSF (black) and after D-AP5 (gray). **(B)** Peak EPSC is reduced after D-AP5 application (*p* = 9.3E-3, Wilcoxon rank-sum test, *n* = 12). **(C)** EPSC 20–80% rise time is not significantly changed after D-AP5 application. **(D)** EPSC decay time is significantly reduced by D-AP5 (weighted tau calculated from two-exponential fit to decay phase of EPSC; *p* = 4.9E-4, Wilcoxon rank-sum test, *n* = 12). **(E)** Example traces showing paired-pulse responses at different intervals before (black) and after D-AP5 (gray). **(F)** No difference in PPR measurements was detected between control (black) and D-AP5 (gray) conditions [Friedman test statistic = 11.9 (15.5), *p* = 0.21, *n* = 11]. *Indicates *p* < 0.05; ns indicates not significant.

Synaptic currents were measured using whole-cell patch clamp recordings from visually identified pyramidal neurons in L2/3 in acute slices of primary somatosensory (barrel) cortex from young (postnatal day 11–22) mice. Electrical stimulation of L4 in the same barrel column using a bipolar theta glass electrode led to a fast, inward current after a brief delay when holding the postsynaptic neuron at −78 mV (Figure 1A).

**FIGURE 2:**
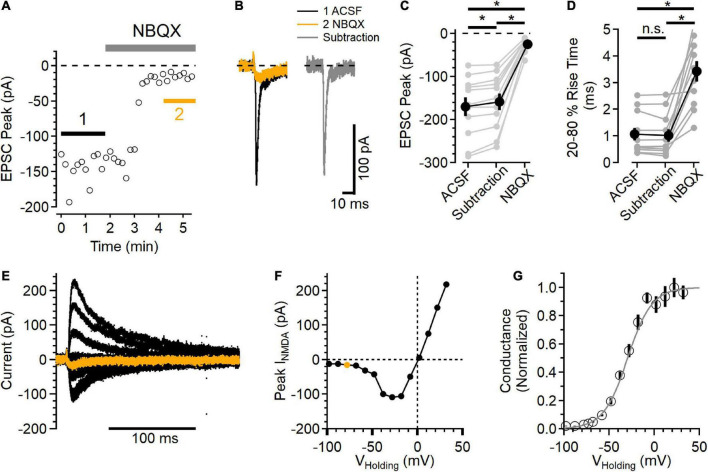
Postsynaptic NMDAR currents in layer 2/3 neurons. **(A)** Time course of NBQX (10 μM) inhibition of peak EPSC current measured in voltage clamp, *V*_Holding_ = –78 mV. **(B)** Average EPSCs from the regions indicated in **(A)**. The black trace is the control EPSC (ACSF), the orange trace is after NBQX addition, and the gray trace is the subtraction of the NBQX trace from the ACSF trace. **(C)** Peak NBQX-sensitive current isolated by subtraction is smaller than the peak ACSF control trace, and the current remaining in NBQX was significantly smaller than either the ACSF or subtracted condition [Friedman test statistic = 22.0 (6.0), *p* = 1.7E-5, ACSF vs. subtracted current *p* = 9.8E-4, ACSF vs. NBQX *p* = 9.8E-4, and subtracted vs. NBQX *p* = 9.8E-6, Wilcoxon rank-sum tests, *n* = 11]. **(D)** The 20–80% rise time of the NBQX-sensitive synaptic current was not significantly different from control, while currents measured in NBQX had a significantly slower rise time [Friedman test statistic = 16.5 (6.0), *p* = 2.6E-4, 20–80% rise time in NBQX was 3.44 ± 0.38 ms, compared to 1.06 ± 0.24 ms in control ACSF, *p* = 9.8E-4, and 1.02 ± 0.24 ms in the subtracted traces, *p* = 9.8E-4 compared to control, Wilcoxon rank-sum tests, *n* = 11]. **(E)** A family of NMDAR currents was measured in the presence of NBQX at different holding potentials, *V*_Holding_ = –78 mV in orange. **(F)** NMDAR I-V curve was constructed by measuring the peak NMDAR current at each holding voltage tested. The peak measurement of the orange trace in **(E)** is shown as an orange symbol. **(G)** Normalized NMDAR conductance-voltage relationship (symbols are mean ± SEM from *n* = 11 recordings). The light gray trace is a fit to the equation *f*(*V*)1/[1 + 0.11 exp(–0.074*V)], which has a midpoint at –29.5 mV. *Indicates *p* < 0.05; ns indicates not significant.

**FIGURE 3:**
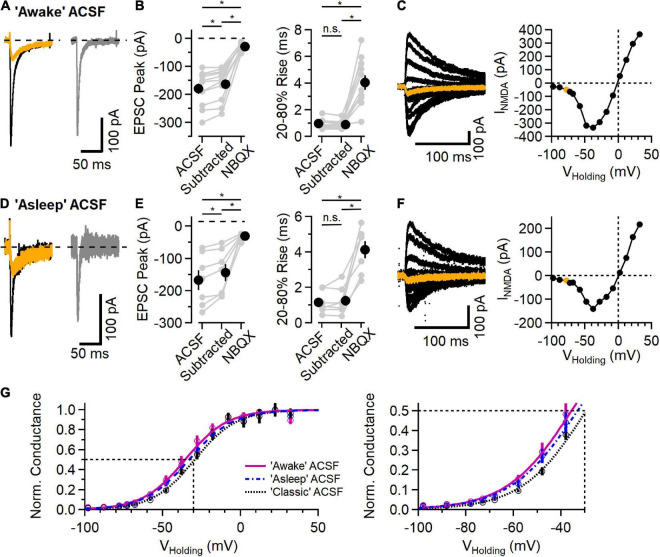
NMDAR component of the EPSC in L2/3 neurons in physiological ionic conditions. **(A)** EPSC measured in “awake” ACSF (1.2 mM Ca^2+^/0.7 mM Mg^2+^/4.2 mM K^+^), before (black trace) and after addition of 10 μM NBQX (orange trace), and the subtracted (NBQX-sensitive) AMPAR component (gray trace). **(B)** Left, NBQX-subtracted traces show a significant contribution of NMDAR current to EPSC peak measurement at a holding potential of –78 mV [Friedman test statistic = 21.0 (6.6), *p* = 2.7E-5; comparing ACSF condition versus subtracted condition, *p* = 2.0E-3, comparing ACSF versus NBQX condition, *p* = 9.8E-4, comparing subtracted condition versus NBQX condition, *p* = 9.8E-4, Wilcoxon rank-sum test, *n* = 11]. Right, 20–80% rise-time measurements of the synaptic current are significantly slower in NBQX [Friedman test statistic = 17.2 (6.6), *p* = 1.8E-4; comparing ACSF condition versus subtracted condition, *p* = 0.07 (n.s., not significant), comparing ACSF versus NBQX condition, *p* = 9.8E-4, comparing subtracted condition versus NBQX condition, *p* = 9.8E-4, Wilcoxon rank-sum test, *n* = 11]. **(C)** A Family of synaptic NMDAR currents measured at different holding potentials (each trace is an average of 5 trials), with the trace measured at –78 mV highlighted in orange. Right, peak current-voltage relationship from this example cell. **(D–F)** Similar to **(A–C)** except measured in “asleep” ACSF (1.3 mM Ca^2+^/0.8 mM Mg^2+^/3.8 mM K^+^). **(D)** Example EPSCs measured in ACSF (black) and NBQX (orange), and the subtracted AMPAR component (gray). **(E)** Isolated AMPAR EPSC peak measurement is smaller than control, and a significant synaptic current remains in NBQX [Friedman test statistic = 14.0 (7.2), *p* = 9.1E-4; comparing ACSF condition versus subtracted condition, *p* = 1.6E-2, comparing ACSF versus NBQX condition, *p* = 1.6E-2, comparing subtracted condition versus NBQX condition, *p* = 1.6E-2, Wilcoxon rank-sum test, *n* = 7]. The 20–80% rise time is longer in NBQX [Friedman test statistic = 11.1 (7.2), *p* = 3.2E-4; comparing ACSF condition versus subtracted condition, *p* = 0.93 (n.s., not significant), comparing ACSF versus NBQX condition, *p* = 1.6E-2, comparing subtracted condition versus NBQX condition, *p* = 1.6E-2, Wilcoxon rank-sum test, *n* = 7]. **(F)** An example NMDAR current family at different holding potentials and the peak current-voltage relationship (the trace measured at –78 mV is highlighted in orange). **(G)** Normalized NMDAR conductance (Mean ± SEM) versus voltage curves from recordings in “awake” ACSF (fuchsia, *N* = 11), “asleep” ACSF (blue symbols and dashed line, *n* = 7), and “classic” ACSF (2.0 mM Ca^2+^/1.0 mM Mg^2+^/2.5 mM K^+^; data from Figure 2, black symbol and dotted line, *n* = 11). Smooth curves are fits to the function *f*(*V*)=1/[1 + a*exp(b*V)]: “awake” ACSF (fuchsia) was fit with *a* = 0.073 and *b* = –0.074, with a midpoint at –35.6 mV; “asleep” ACSF (blue) was fit with *a* = 0.095 and *b* = –0.071, with a midpoint at –33.1 mV; and “classic” ACSF (gray) was fit with *a* = 0.11 and *b* = –0.074, with a midpoint at –29.5 mV. *Indicates *p* < 0.05; ns indicates not significant.

Application of the NMDAR antagonist D-AP5 (50 μM) led to a slight decrease in the peak synaptic current (Figure 1B, after D-AP5 current was reduced to 82.4 ± 4.6% of the baseline, *p* = 9.3E-3, *n* = 12, Wilcoxon rank-sum test). NMDAR inhibition with D-AP5 did not affect the rise time of the EPSC (Figure 1C, 20–80% rise time was 1.00 ± 0.12 in the baseline period and 1.24 ± 0.315 after D-AP5, *p* = 0.91, *n* = 12, Wilcoxon rank-sum test). The time course of EPSC decay, measured using the weighted tau from a two-exponential fit to the falling phase of the EPSC, was significantly faster after D-AP5 application (Figure 1D, weighted tau was reduced from 15.95 ± 3.37 ms in ACSF to 5.68 ± 3.37 ms in D-AP5, *p* = 4.9E-4, *n* = 12, Wilcoxon rank-sum test).

These effects of D-AP5 on the EPSC time course indicate that NMDARs are active in the postsynaptic neuron at this holding potential, contribute to the peak EPSC current, and prolong the synaptic current. The rising phase of the EPSC was not changed by D-AP5, consistent with AMPAR current activating first and providing most of the fast synaptic current, followed by the slower activation of NMDARs, similar to previous recordings from L4-L2/3 synapses (e.g., Feldman, 2000; Feldmeyer et al., 2002).

We also measured short-term plasticity in these synapses using a paired-pulse paradigm at different intervals to test for presynaptic effects of D-AP5 (Brasier and Feldman, 2008). An example paired-pulse recording is shown in Figure 1E in ACSF (black traces, top) and after D-AP5 application (gray traces, bottom). The paired-pulse ratio (PPR), measured as the amplitude of the second response relative to the first, shows a time course of short-term plasticity similar to what has been reported at this synapse type (Figure 1F; Castro-Alamancos and Connors, 1997). In these experiments, D-AP5 did not affect PPR [*n* = 11, Friedman test statistic = 11.9 (15.5), *p* = 0.21].

### Postsynaptic NMDA receptor-mediated currents

In the slice preparation, bath application of D-AP5 inhibits NMDARs in all neurons, both the postsynaptic neuron being recording as well as presynaptic neurons that are being stimulated. The NMDAR component in postsynaptic neurons is often assumed to be negligible at negative holding potentials due to the voltage-dependent Mg^2+^ block of the NMDAR ion channel (e.g., Sjöström et al., 2003; Bender et al., 2006), coupled with the slower intrinsic kinetics of NMDAR activation compared to AMPARs (Feldmeyer et al., 2002). Several studies have focused on the role of NMDARs contributing to presynaptic release properties (e.g., Brasier and Feldman, 2008; Christie and Jahr, 2008; Banerjee et al., 2009; Larsen et al., 2011). However, the measurable NMDAR component in EPSCs (Figure 1) near the neuronal resting potential raises the question of whether postsynaptic NMDAR current can contribute to the synaptic response over a wider range of membrane potentials than typically appreciated.

To test the voltage range of postsynaptic NMDAR current, NMDAR currents were isolated by blocking AMPARs with 10 μM NBQX. GABA_*A*_Rs were blocked with 50 μM picrotoxin. Figure 2A shows an example of NBQX inhibition of the EPSC peak. The synaptic currents measured before (black trace) and after NBQX application (orange trace) were then subtracted to define the AMPAR component (gray trace) of the EPSC (Figure 2B). In every experiment, there was a measurable inward synaptic current remaining in the presence of NBQX (−24.4 ± 4.7 pA), and the peak of the isolated AMPAR component was smaller than the EPSC in control conditions [Figure 2C, subtracted peak EPSC was 93.7 ± 0.1% of control, Friedman test statistic = 22.0 (6.0), *p* = 1.7E-5, ACSF versus subtracted current *p* = 9.8E-4, ACSF vs. NBQX *p* = 9.8E-4, and subtracted versus NBQX *p* = 9.8E-6, Wilcoxon rank-sum tests, *n* = 11], indicating that the NMDAR current did contribute to the peak EPSC at this voltage.

The current remaining in NBQX had slower kinetics than the control EPSC as well as the AMPAR component isolated by subtraction [Figure 2D, Friedman test statistic = 16.5 (6.0), *p* = 2.6E-4; 20–80% rise time in NBQX was 3.44 ± 0.38 ms, compared to 1.06 ± 0.24 ms in control ACSF, *p* = 9.8E-4, and 1.02 ± 0.24 ms in the subtracted traces, *p* = 9.8E-4 compared to control, Wilcoxon rank-sum tests, *n* = 11]. In a subset of these experiments (7/11), D-AP5 was subsequently added, inhibiting the remaining synaptic current.

The voltage-dependence of the synaptic NMDAR currents was then measured over a wide range of holding potentials (Figure 2E) to define the NMDAR current-voltage relationship. Figure 2F shows the peak NMDAR current as a function of holding voltage. The synaptic NMDAR reversal potential in these conditions was 0.02 ± 1.00 mV (*n* = 11), estimated using a linear fit between the two measurements that straddled current reversal. Using this estimated reversal potential, the normalized synaptic NMDAR conductance was calculated (Jahr and Stevens, 1990b) and plotted as a function of holding voltage (Figure 2G). In these conditions, the NMDAR conductance could be well fit with a sigmoid relationship (Jahr and Stevens, 1990b), with a midpoint at −29.5 mV.

### NMDA receptor currents in physiological ionic conditions

Recent measurements of the extracellular ionic environment showed a range of concentrations of Ca^2+^, Mg^2+^, and K^+^ that varied between awake, asleep and anesthetized behavioral states (Ding et al., 2016). Because neuronal activity in general and NMDARs in particular are sensitive to these ions, we measured synaptic responses using ionic conditions mimicking those measured *in vivo* (Ding et al., 2016). Figures 3A–C shows example recordings using ACSF mimicking that measured in awake animals (1.2 mM Ca^2+^, 0.7 mM Mg^2+^, and 4.2 mM K^+^). Synaptic responses in this “awake” ACSF from a holding potential of −78 mV are shown in Figure 3A. As before, the AMPAR component was blocked with NBQX and, in every case, there remained an NMDAR current in response to synaptic stimulation (Figures 3A,B). Figure 3C shows a family of NMDAR mediated responses to synaptic stimulation while holding at potentials between −98 and +32 mV, and a plot of peak response vs. holding potential. In these conditions, the reversal potential of NMDAR current was −0.1 ± 0.7 mV (*n* = 11).

Figures 3D–F shows a similar set of experiments using an ACSF that mimics “asleep” conditions (1.3 mM Ca^2+^, 0.8 mM Mg^2+^, and 3.8 mM K^+^, Ding et al., 2016). In every experiment (*n* = 7), there were measurable NMDAR currents at every voltage tested. In “asleep” ACSF, the reversal potential of NMDAR currents was 0.3 ± 0.4 mV, similar to that in the other ionic conditions.

Figure 3G shows the normalized NMDAR conductance versus voltage for the three ionic conditions, fit with a sigmoid function. As expected, the voltage dependence of the NMDAR conductance is shifted toward more negative voltages in conditions with lower extracellular Mg^2+^, ranging from −29.5 mV in “classic” ACSF (2.0 mM Ca^2+^, 1.0 mM Mg^2+^, and 2.5 mM K^+^) to −35.6 mV in “awake” ACSF.

### NMDA receptor currents in hippocampal Schaffer collateral-CA1 synapses

**FIGURE 4:**
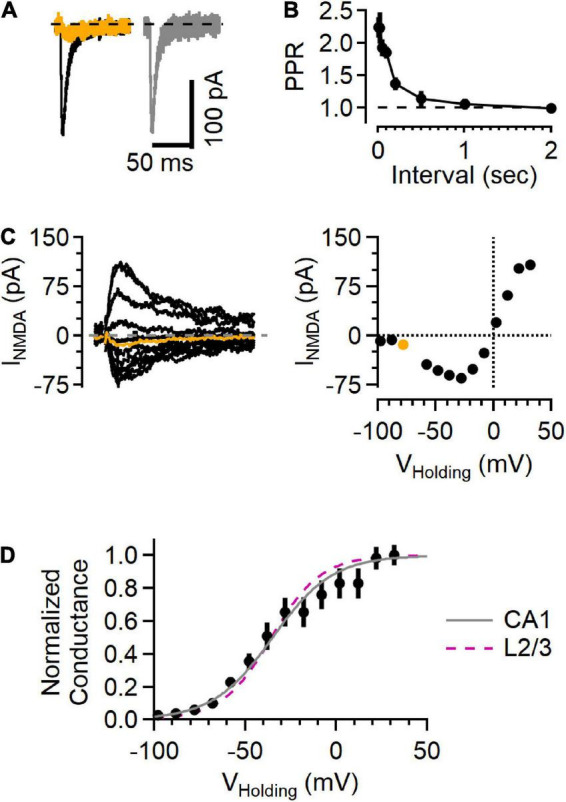
NMDAR responses in Schaffer collateral synapses onto hippocampal CA1 cells show a voltage sensitivity similar to cortical L2/3 cells. **(A)** Example traces recorded from a hippocampal CA1 neuron upon stimulating Schaffer collateral inputs. Left, EPSC in “awake” ACSF (black) and after NBQX application (orange). Right, subtracted (NBQX-sensitive) AMPAR component of the EPSC (gray). **(B)** Paired-pulse response shows large facilitation at short intervals, typical of this synapse type. **(C)** Left, a family of NMDA current responses at different voltages and the current voltage relationship (right), with the measurement while holding at –78 mV highlighted in orange. **(D)** Normalized conductance versus voltage from 5 recordings (black symbols, Mean ± SEM). Gray line is a fit to these data (*a* = 0.12, *b* = –0.061, with the midpoint –35.0 mV), and the fit from the L2/3 recordings in awake ACSF (from Figure 3) is shown as a dashed fuchsia line for comparison.

The NMDAR currents in L4-L2/3 synapses were present at all voltages tested, raising the question of whether this response pattern is unique to this synapse type or whether it might be a general property of synaptic NMDARs. To test this, a similar set of recordings were made in hippocampal CA1 neurons while stimulating Schaffer collateral inputs using “awake” ACSF extracellular solution (Figure 4). These synapses showed short-term facilitation in response to paired-pulse stimulation measured at different intervals (Figure 4B), typical of Schaffer collateral-CA1 synapses (e.g., Jackman et al., 2016), contrasting with the paired-pulse response in the L4-L2/3 synapses (Figure 1F). However, similar to measurements from cortical L4-L2/3 synapses, these hippocampal recordings showed detectable NMDAR current at every voltage tested (Figure 4C). The normalized conductance-voltage relationship was very similar to the L4-L2/3 measurements (Figure 4D), with a midpoint from the fit at −35.0 mV (compared to −35.6 mV in the cortical measurements).

NMDAR currents were smaller in the hippocampal synapses than those in L4-L2/3 synapses. Because the size of the EPSC depends on factors that vary from recording to recording (e.g., number of fibers activated by extracellular stimulation), we used the NMDAR current amplitude measured at +32 mV normalized to the AMPAR current measured at −78 mV (i.e., the N/A ratio) to compare results from the two regions. The N/A ratio in cortical L4-L2/3 neurons was 1.22 ± 0.15 (*n* = 11) and 0.71 ± 0.15 in hippocampal neurons (*n* = 8, *p* = 4.1E-2, Mann–Whitney *U* test). In contrast, NMDAR currents from the different cell types decayed with the same time course (measured as the weighted tau from a two-exponential fit to the falling phase of the synaptic current recorded at +32 mV; 96.9 ± 25.8 ms in L2/3 versus 78.2 ± 35.7 ms in CA1, *p* = 0.15, Mann–Whitney *U* test). These results indicate that the NMDAR currents likely arise from NMDARs composed of similar subunits (Hansen et al., 2021), and that the difference in N/A ratio is therefore likely due to a difference in the number of NMDAR receptors present relative to the number of AMPARs in a given synapse.

### Synaptic Ca^2+^ signals persist with AMPA receptors inhibited

**FIGURE 5:**
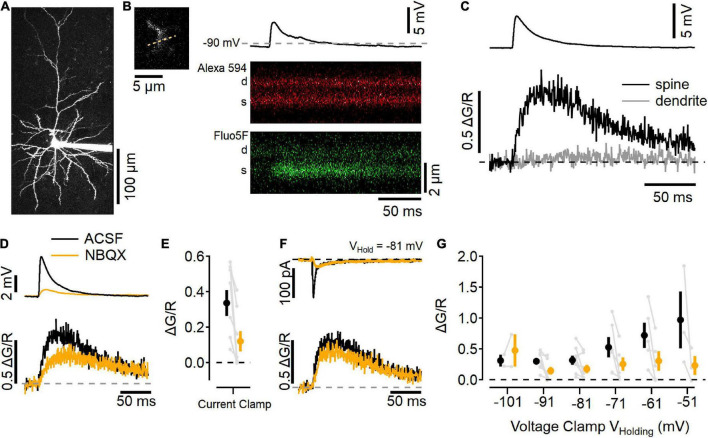
Imaging Ca^2+^ influx at individual L2/3 synaptic contacts. **(A)** Two-photon laser scanning image of a L2/3 neuron filled with 10 μM Alexa 594 (maximum projection image). **(B)** Left, a dendritic segment and spine that was active after electrical stimulation in L4 was scanned repeatedly to generate line scan images, right. Example trial of a current-clamp recording (resting potential near –90 mV) with an EPSP elicited by stimulation (top), and line scans in the red channel (Alexa 594; middle), and green channel (Fluo-5F, 300 μM; bottom) showing an increase in Ca^2+^-sensitive green fluorescence in the spine (s), but not the parent dendrite (d). **(C)** Image quantification of the synaptic response showing the change in green fluorescence from the baseline level before simulation, relative to the red fluorescence (ΔG/R, traces are an average of 10 trials). **(D)** Current-clamp measurements in “awake” ACSF (black) and after 10 μM NBQX (orange) show NBQX reduces the EPSP (top) as well as Ca^2+^ influx into the spine (bottom). **(E)** Summary of eight current-clamp recordings before and after NBQX application (individual experiments are shown in gray, black symbol is mean ± SEM in ACSF, and the orange symbol is the mean ± SEM in NBQX). **(F)** Example voltage-clamp recording holding at –81 mV shows synaptic currents (top) and Ca^2+^ transients (bottom) before (black) and after (orange) NBQX application. **(G)** Summary of synapse imaging experiments performed in voltage clamp at different holding potentials [*N* = 2–8 recordings at each voltage, symbols as in **(E)**].

Electrophysiological recording of synaptic activity gives the overall response to stimulation, which represents the synchronous activity of multiple synapses. Each L4 axon makes on average ∼5 synaptic contacts onto a given L2/3 neuron (Silver et al., 2003), and electrical stimulation likely recruits multiple L4 axons. To test whether the NMDAR activity leads to measurable Ca^2+^ influx at individual synaptic contacts, we used two-photon laser scanning microscopy to image Ca^2+^-sensitive fluorescence in dendritic processes of L2/3 neurons in physiological ionic conditions (Figure 5).

**FIGURE 6:**
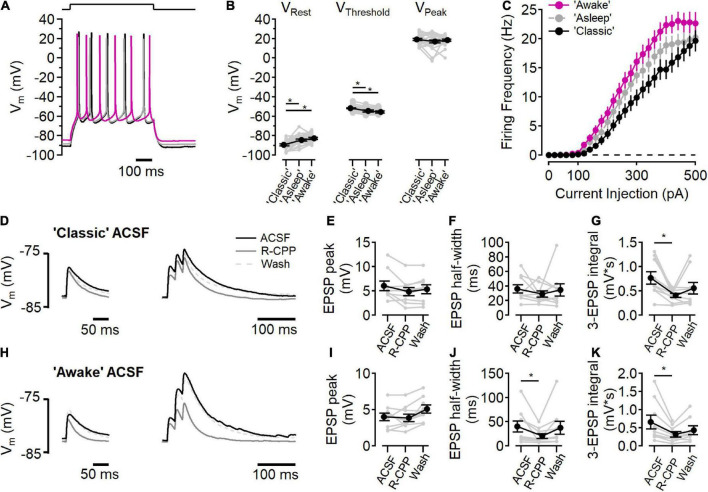
NMDARs contribute to synaptic integration. **(A–C)** Current clamp recordings in “awake” (fuchsia), “asleep” (gray), and “classic” ACSF conditions. **(A)** Example voltage traces in each condition in response to a current step to stimulate action potential firing. **(B)** Measurements of resting membrane potential (V_*Rest*_), action potential threshold (V_*Threshold*_), and the peak voltage of the action potential (V_*Peak*_) in each ACSF condition [for V_*Rest*_, Friedman test statistic = 25.4 (6.0), *p* = 3.0E-6; “classic” versus “asleep” *p* = 1.6E-5, “classic” versus “awake,” *p* = 3.7E-7, Wilcoxon rank-sum test; for V_*Threshold*_, Friedman test statistic = 28.1 (6.0), *p* = 8.0E-7; “classic” versus “asleep” *p* = 1.9E-5, “classic” versus “awake,” *p* = 3.0E-8, Wilcoxon rank-sum test; for V_*Peak*_ Friedman test statistic = 0.2 (6.0), *p* = 0.89; *N* = 27]. **(C)** Action potential firing frequency as a function of current injection. **(D–G)** Peak EPSP response to a single stimulation (**D**, left) and three stimuli at 50 Hz (**D**, right) in “classic” ACSF (black traces), with 10 μM R-CPP (solid gray trace), and after washing R-CPP (gray dashed traces). **(E)** EPSP peak amplitude in “classic” ACSF. **(F)** EPSP half-width in “classic” ACSF. **(G)** R-CPP decreases the integrated response to three stimuli [Friedman test statistic = 10.9 (6.3), *p* = 2.9E-3; comparing ACSF versus R-CPP, *p* = 3.9E-3, Wilcoxon rank-sum test; *N* = 9]. **(H–K)** Peak EPSP response to a single stimulation (**H**, left) and three stimuli at 50 Hz (**H**, right) in “awake” ACSF (black traces), with 10 μM R-CPP (solid gray trace), and after washing R-CPP (gray dashed traces). **(I)** EPSP peak amplitude in “awake” ACSF. **(J)** EPSP half-width is reduced by R-CPP in “awake” ACSF [Friedman test statistic = 10.9 (6.3); comparing ACSF versus R-CPP, *p* = 3.9E-3; *N* = 9]. **(K)** R-CPP decreases the integrated response to three stimuli in “awake” ACSF [Friedman test statistic = 12.3 (6.3), *p* = 2.6E-4, comparing ACSF versus R-CPP, *p* = 7.8E-3, Wilcoxon rank-sum test; *N* = 9]. *Indicates *p* < 0.05.

Figure 5A shows a maximum-projection image of a L2/3 neuron that had been filled with 10 μM Alexa 594 and 300 μM Fluo-5F via the recording pipette. Synaptic responses were found by searching the dendritic arbor for Ca^2+^-sensitive fluorescence increases while stimulating in L4 (Figure 5B; Padamsey et al., 2019). This example neuron rested near −90 mV, and responded to L4 stimulation with a ∼5 mV depolarization and a Ca^2+^ increase in the dendritic spine but not the adjacent dendrite (Figure 5C). Figure 5D shows this same spine before (black traces) and after NBQX application (orange traces). NBQX decreased the EPSP size and reduced, but did not eliminate, the Ca^2+^ influx into the spine, similar to uncaging-evoked responses in L2/3 dendritic spines (Landau et al., 2022), as well as stimulated responses in L4 dendritic spines (Nevian and Sakmann, 2004). Figure 5E shows a summary of eight experiments in which NBQX was added. In these experiments, responses were also tested in voltage clamp while holding at different potentials (Figures 5F,G). NBQX reduced but did not eliminate the synaptic Ca^2+^ influx over a wide range of potentials.

### NMDA receptors contribute to synaptic integration

Changes in the extracellular ionic composition can influence the intrinsic electrical properties of cells, in addition to affecting NMDARs directly. To measure the intrinsic cell properties in L2/3 neurons, responses to current steps (500 ms, from −100 to +500 pA, 20 pA increments) were measured in each of the three ACSF solutions (Figure 6A). In “classic” ACSF, resting membrane potential was more hyperpolarized than in either “awake” or “asleep” ACSF [Figure 6B, “classic”: −89.4 ± 0.9 mV, “asleep”: −84.5 ± 0.9 mV, “awake”: −82.7 ± 1.0 mV; Friedman test statistic = 25.4 (6.0), *p* = 3.0E-6; “classic” versus “asleep” *p* = 1.6E-5, “classic” versus “awake,” *p* = 3.7E-7, Wilcoxon rank-sum test, *n* = 27 cells]. AP firing threshold occurred at more a depolarized potential in “classic” ACSF than “awake” or “asleep” ACSF [Figure 6B, “classic”: −51.6 ± 0.5 mV, “asleep”: −54.7 ± 0.9 mV, “awake”: −55.6 ± 1.1 mV; Friedman test statistic = 28.1 (6.0), *p* = 8.0E-7; “classic” versus “asleep” *p* = 1.9E-5, “classic” versus “awake,” *p* = 3.0E-8, Wilcoxon rank-sum test, *n* = 27 cells]. The peak of the action potential was similar in all three solutions [18.2 ± 0.7 mV; Friedman test statistic = 0.2 (6.0), *p* = 0.89]. Figure 6C shows the action potential firing frequency as a function of current injection in each ACSF condition. Integration of the number of action potentials in each cell in each ACSF condition showed an increase in action potentials fired in “asleep” and in “awake” relative to the “classic” ACSF [Friedman test statistic = 17.7 (6.0), *p* = 1.4E-4; “classic” versus “asleep” *p* = 2.6E-2, “classic” versus “awake”, *p* = 2.1E-4, “asleep” versus “awake,” *p* = 1.9E-2, Wilcoxon rank-sum test, *n* = 27 cells], consistent with increased excitability when divalent ion concentrations are lowered (Hille et al., 1975).

The presence of measurable synaptic NMDAR activity at all voltages tested (Figures 1–5), raised the question whether tonic NMDAR activation could contribute to the resting membrane potential (Sah et al., 1989). To test this, the effect of D-AP5 on resting membrane potential was measured in each ACSF condition and no hyperpolarization was seen (“classic” *p* = 0.99, “asleep,” *p* = 0.92, “awake,” *p* = 0.97, Wilcoxon rank-sum test, *n* = 27 cells), indicating that there is little basal NMDAR activation at rest, consistent with a very low tonic extracellular glutamate concentration (Cavelier and Attwell, 2005; Herman and Jahr, 2007; Chiu and Jahr, 2017).

We next measured the contributions of NMDARs to synaptic activation in current clamp experiments in “classic” ACSF (Figures 6D–G) and “awake” ACSF (Figures 6H–K). In “classic” ACSF, there was no effect of NMDAR inhibition (by 10 μM R-CPP) on a single EPSP peak (Figure 6E), or half-width (Figure 6F), however, in response to three stimuli at 50 Hz, R-CPP significantly reduced the integrated voltage change (Oberle et al., 2022), indicating a functional role of NMDARs in the integration of synaptic signaling. In “awake” ACSF (Figures 6H–K), R-CPP did not reduce the peak of a single EPSP (Figure 6I), but did decrease the half-width of the EPSP [Figure 6J; Friedman test statistic = 10.9 (6.3), *p* = 2.9E-3; R-CPP versus control *p* = 3.9E-3, Wilcoxon rank-sum test, *n* = 9]. Additionally, R-CPP reduced the three EPSP integral [Figure 6K; Friedman test statistic = 12.3 (6.3), *p* = 2.6E-4; R-CPP versus control *p* = 7.8E-3, Wilcoxon rank-sum test, *n* = 8].

The initial response is smaller in the “awake” ionic condition (6.2 ± 0.9 mV in “classic” versus 4.1 ± 0.5 mV in “awake” ACSF; *p* = 0.02, Wilcoxon rank-sum test, *n* = 9), likely due to reduced Ca^2+^ (1.2 mM in “awake” ACSF versus 2.0 mM in “classic” ACSF) lowering presynaptic release probability (Dodge and Rahamimoff, 1967). Although the EPSP was smaller, the R-CPP effect on half-width of a single EPSP in a physiological ionic environment shows that NMDARs contribute to synaptic potentials, even at voltages near the resting membrane potential.

## Discussion

### Postsynaptic NMDA receptors respond to single stimuli

NMDA receptors are often thought of as coincidence detectors because simultaneous ligand binding and depolarization lead to NMDAR activation and relief of voltage-dependent Mg^2+^ block. This non-linear enhancement of synaptic Ca^2+^ influx (e.g., Sabatini et al., 2002; Nevian and Sakmann, 2004) underlies glutamatergic synaptic plasticity signaling in many cell types (Malenka and Nicoll, 1993; Feldman, 2012). Coincidence detection is particularly important for synaptic plasticity signaling in cortical L2/3 neurons, where back-propagating action potentials depolarize the dendritic membrane, leading to a large Ca^2+^ influx in synapses with glutamate-bound NMDARs (Nevian and Sakmann, 2006). Many postsynaptic factors contribute to this signaling mechanism, including the ability of the dendrites to support action potential back-propagation (Nevian and Sakmann, 2004) and the local dendritic morphology (Landau et al., 2022).

In our experiments, in both L4-L2/3 synapses as well as hippocampal Schaffer collateral-CA1 synapses, we found that postsynaptic NMDARs pass current at all voltages, even with synaptic AMPARs blocked. This observation shows that, rather than being an all-or-none switch depending on temporal coincidence of other depolarizing factors, NMDARs alone can contribute to synaptic function even in the case of isolated synaptic events. NMDAR-dependent Ca^2+^ influx at subthreshold voltages has been previously observed in dendritic spines after local stimulation in CA1 neurons (Sabatini et al., 2002) and cortical L2/3 neurons (Nevian and Sakmann, 2006), as well as L2/3 dendritic spines in response to focal two-photon glutamate uncaging (Landau et al., 2022).

Indeed, NMDAR currents that have been reported in miniature synaptic events in cortical L4 neurons (Espinosa and Kavalali, 2009). The NMDAR component detected in single spines (Figure 5) likely corresponds to single vesicle release events (Silver et al., 2003). If spine NMDARs signal more regularly than previously assumed, it is possible that evoked release could serve a similar functional role as spontaneous release in maintaining dendritic signaling integrity (Kavalali, 2020).

While NMDAR-mediated Ca^2+^ influx is likely to participate in intracellular signaling, our results show that NMDARs can also contribute to integration of synaptic signals via EPSPs. This could influence the functional integration of ongoing synaptic activity, as it does in cortical L5 neurons (Larkum et al., 2009), and inferior colliculus (Oberle et al., 2022).

### NMDA receptor activity is enhanced in physiological ionic conditions

NMDA receptors are sensitive to postsynaptic voltage due to extracellular Mg^2+^ ions inhibiting current flux through the NMDAR ion channel (Mayer et al., 1984; Nowak et al., 1984). Recent measurements of the ionic composition of interstitial fluid *in vivo* showed that Mg^2+^ is regulated in different behavior states, and ranges from ∼0.7 to 1.3 mM (Ding et al., 2016; Rasmussen et al., 2020). We found measurable NMDAR activity in all ionic conditions tested. In conditions mimicking those measured in awake animals, the NMDAR voltage dependence was shifted by ∼6 mV, which led to an increase in NMDAR current activity at hyperpolarized potentials.

Measurements of synaptic response in the “awake” ionic environment result in counterintuitive synaptic processing outcomes. The reduced Ca^2+^ would be expected to decrease synaptic release (Dodge and Rahamimoff, 1967) and does disrupt NMDAR-dependent synaptic plasticity in hippocampal slices (Inglebert et al., 2020; Inglebert and Debanne, 2021). Although physiological ionic concentrations lead to decreased EPSP magnitudes, the NMDAR component amplified the response (Figure 6). Because the importance of spike-timing dependent plasticity in developing L4-L2/3 synapses (Feldman and Brecht, 2005; Feldman, 2012), it will be interesting to test how the physiological ionic environment alters the synaptic plasticity rules at this synapse.

### Experimental control of membrane voltage

In voltage clamp experiments, voltage control and subsequent synaptic current measurements can be greatly distorted both as a function of distance from the recording pipette (Williams and Mitchell, 2008), as well as by the electrical isolation created by the spine geometry (Svoboda et al., 1996; Beaulieu-Laroche and Harnett, 2018; Cornejo et al., 2022). Estimates of the voltage drop between an active spine with AMPARs intact and the recording electrode have ranged from ∼3 mV in CA1 neurons (Svoboda et al., 1996) to >50 mV in L5 neurons (Beaulieu-Laroche and Harnett, 2018).

The degree of electrical isolation of synaptic connections between L4 and L2/3 has not been systematically examined; however, several observations indicate that synaptic currents recorded in voltage clamp can give some useful readout of synaptic receptor function. Electron microscopy reconstructions of L4-L2/3 synapses show a distribution that is relatively close to the soma (Feldmeyer et al., 2002) and often occur on the dendrite itself (Silver et al., 2003). The rapid kinetics and large size of AMPAR currents are the main source of glutamatergic synaptic conductance that would distort voltage clamp of the synaptic compartment (Beaulieu-Laroche and Harnett, 2018); however, the voltage clamp experiments testing the NMDAR voltage dependence were done with AMPARs blocked (Figures 2–5). It is possible that the NMDAR currents themselves could be large enough to locally depolarize the synaptic compartment, but this would imply that the currents are able to generate a considerably large response in the first place, which is our conclusion. The NMDAR current-voltage relationships measured here (Figures 2–4) reverse near 0 mV, as expected from NMDAR channels. The voltage dependence due to Mg^2+^ block also showed a similar slope to that measured in more reduced preparations [e.g., cultured mouse neurons (Nowak et al., 1984), dissociated mouse spinal cord neurons (Mayer et al., 1984), and cultured rat hippocampal neurons (Jahr and Stevens, 1990b), which in turn matched NMDAR behavior measured in single channel recordings (Jahr and Stevens, 1990a)].

### NMDA receptor subunit composition

NMDA receptors form as heterotetramers consisting of two obligate GluN1 (glycine/D-serine binding) subunits and two subunits from either the GluN2 family (the glutamate-binding 2A, 2B, 2C, 2D subunits) or the GluN3 family (the glycine-binding 3A and 3B subunits), either as diheteromers or triheteromers (Paoletti et al., 2013; Hansen et al., 2021). The specific combination of subunits in a given receptor determines the biophysical properties, the response to ligands, as well as selective pharmacology (Hansen et al., 2018). Cortex and hippocampus primarily express the GluN1, GluN2A, and GluN2B subunits (Monyer et al., 1992). In embryonic and early postnatal development, functional NMDARs in these regions are primarily GluN2B-containing diheteromers, with GluN2A subunit expression increasing over time (Monyer et al., 1994), and functional triheteromers likely to be the dominant form at mature hippocampal synapses (Gray et al., 2011; Tovar et al., 2013). GluN2C, 2D, and 3A containing NMDARs are less sensitive to voltage-dependent Mg^2+^ block (Hansen et al., 2021), raising the possibility that receptors containing these subunits underlie the currents measured in the experiments presented here. Previous studies examining the L4-L2/3 synapse have found evidence for GluN2C/GluN2D (Binshtok et al., 2006; Banerjee et al., 2009) and GluN3A (Larsen et al., 2011) in L4 but not the postsynaptic L2/3 neurons. While we did not explicitly test for different subunit compositions, and cannot rule out a contribution of these receptor subtypes, the kinetics of the synaptic NMDAR currents in our cortical and hippocampal recordings were similar, and in a range consistent with GluN2A/2B triheteromeric receptors (Tovar et al., 2013; Hansen et al., 2014; Sun et al., 2017).

### Location of synaptic NMDA receptors

These experiments were designed to test the function of postsynaptic NMDARs. Dissecting the synaptic currents showed both AMPAR and NMDAR components combined to form the overall EPSC, and the pharmacologically isolated currents had kinetic properties similar to each receptor type (Feldmeyer et al., 2002). Previous studies have concluded that non-postsynaptic NMDARs also contribute to the synaptic responses at L4-L2/3 synapses (Bender et al., 2006; Nevian and Sakmann, 2006; Brasier and Feldman, 2008; Rodríguez-Moreno and Paulsen, 2008; Rodríguez-Moreno et al., 2013; Neubauer et al., 2022). One line of evidence for this conclusion rests on the assumption that, at voltages near neuronal resting potential, an effect of NMDAR antagonism on synaptic responses to single stimuli does not arise from postsynaptic NMDARs. Synaptic NMDAR current (Figures 2–4), Ca^2+^ influx (Figure 5), and potentials (Figure 6), demonstrate the extent to which NMDARs can signal in the absence of coincident depolarization, calling into question that assumption.

### Summary

Our experiments show that NMDARs can contribute to synaptic signaling at all voltages. NMDAR activity is increased in physiological ionic conditions. This NMDAR activity influences synaptic responses, even in the absence of concurrent depolarization, and provides a Ca^2+^ source local to active synapses, even at voltages near the neuronal resting potential.

## Data availability statement

The original contributions presented in this study are included in the article/supplementary material, further inquiries can be directed to the corresponding author.

## Ethics statement

The animal study was reviewed and approved by the Institutional Animal Care and Ethics Committees of University of Göttingen.

## Author contributions

DC and BC conceived and performed all the experiments, analyzed the data, and wrote and edited the manuscript. Both authors contributed to the article and approved the submitted version.

## Conflict of interest

The authors declare that the research was conducted in the absence of any commercial or financial relationships that could be construed as a potential conflict of interest.

## Publisher’s note

All claims expressed in this article are solely those of the authors and do not necessarily represent those of their affiliated organizations, or those of the publisher, the editors and the reviewers. Any product that may be evaluated in this article, or claim that may be made by its manufacturer, is not guaranteed or endorsed by the publisher.
